# Using whole-genome sequencing data to derive the homologous recombination deficiency scores

**DOI:** 10.1038/s41523-020-0172-0

**Published:** 2020-08-07

**Authors:** Xavier M. de Luca, Felicity Newell, Stephen H. Kazakoff, Gunter Hartel, Amy E. McCart Reed, Oliver Holmes, Qinying Xu, Scott Wood, Conrad Leonard, John V. Pearson, Sunil R. Lakhani, Nicola Waddell, Katia Nones, Peter T. Simpson

**Affiliations:** 1 Centre for Clinical Research, Faculty of Medicine, The University of Queensland, Brisbane, QLD Australia; 2 QIMR Berghofer Medical Research Institute, Brisbane, QLD Australia; 3 Pathology Queensland, Royal Brisbane & Women’s Hospital, Brisbane, QLD Australia

**Keywords:** Breast cancer, Cancer genomics, Breast cancer, Tumour biomarkers, Medical genomics

## Abstract

The homologous recombination deficiency (HRD) score was developed using whole-genome copy number data derived from arrays as a way to infer deficiency in the homologous recombination DNA damage repair pathway (in particular *BRCA1* or *BRCA2* deficiency) in breast cancer samples. The score has utility in understanding tumour biology and may be indicative of response to certain therapeutic strategies. Studies have used whole-exome sequencing to derive the HRD score, however, with increasing use of whole-genome sequencing (WGS) to characterise tumour genomes, there has yet to be a comprehensive comparison between HRD scores derived by array versus WGS. Here we demonstrate that there is both a high correlation and a good agreement between array- and WGS-derived HRD scores and between the scores derived from WGS and downsampled WGS to represent shallow WGS. For samples with an HRD score close to threshold for stratifying HR proficiency or deficiency there was however some disagreement in the HR status between array and WGS data, highlighting the importance of not relying on a single method of ascertaining the homologous recombination status of a tumour.

## Introduction

The identification of tumours that are deficient in the homologous recombination (HR) DNA damage repair pathway is clinically important, as these tumours have been shown to be sensitive to DNA-damaging therapy, such as platinating chemotherapeutic agents, and poly-(ADP-ribose) polymerase-inhibitors (PARP-i)^1,2^. HR deficient tumours have a higher degree of genomic instability represented by a high number of point mutations, chromosomal structural rearrangements and copy number changes. In an effort to develop a biomarker of HR deficiency, Telli et al.^1^ derived the homologous recombination deficiency (HRD) score, which consists of the sum of three independent measures of genomic instability reflecting structural aberrations: (i) the number of sub-chromosomal regions with Loss Of Heterozygosity (LOH) regions >15 Mb (HRD-LOH)^3^; (ii) large scale state transitions (LST), which represents the number of chromosomal breaks between flanking regions of at least 10 Mb^4^; and, (iii) the number of sub-chromosomal regions undergoing allelic imbalance extending to the telomeres (NtAI)^5^. Telli et al.^1^ demonstrated that the combination of the three scores performed best at distinguishing HR deficient from proficient tumours and that high HRD scores predicted HR deficiency and sensitivity to platinating agents. Furthermore, Von Walhde et al.^6^ reported that the HRD score was highly consistent between samples from different areas of the same tumour, making it a useful biomarker of HR deficiency, with low sampling variability. The HRD score was used as an important parameter in the HRDetect tool^7^, which was developed as a predictor of HR status using multiple genomic parameters including mutational signatures derived from whole-genome sequencing (WGS) data.

Most of the work published to date using HRD scores is based on single-nucleotide polymorphism (SNP) array-derived DNA copy number data. Given the rising use of next-generation sequencing (NGS) in the research and clinical context, it is important to characterise how the HRD score derived from NGS-based approaches compare to that derived using an array-based platform. A good correlation between the array- and whole-exome sequencing (WES)-derived overall HRD score (Pearson’s correlation coefficient of 0.87) and the individual components of the HRD score (Pearson’s correlation coefficient of 0.84 for the NtAI, 0.79 for the LST and 0.73 for the HRD-LOH) was shown in a cohort of 139 breast cancer patients^8^. Several other groups have employed WES data to derive the HRD score as an indication of the status of the HR pathway in tumours^9–11^. Here we compare HRD scores calculated from DNA copy number data obtained from arrays, WGS and in downsampled, low coverage WGS data from a cohort of familial breast cancers.

## Results

### Comparing array- and WGS-derived HRD score and HRD score components

SNP array and WGS data was generated from 67 paired normal/tumour samples from a familial breast cancer cohort, including carriers of pathogenic germline mutations in *BRCA1* (*n* = 19) or *BRCA2* (*n* = 20) and 28 tumours from high risk individuals from breast cancer families not attributed to *BRCA1* or *BRCA2* germline mutations (non-*BRCA1/2*)^12^. Genome-wide DNA copy number data was estimated using ASCAT^13^ for the arrays, and ascatNgs^14^ for the WGS data, and the three components of the HRD score (HRD-LOH, LST and NtAI) were derived according to Marquard et al.^15^. The HRD score was calculated through the sum of the three components.

**Fig. 1:**
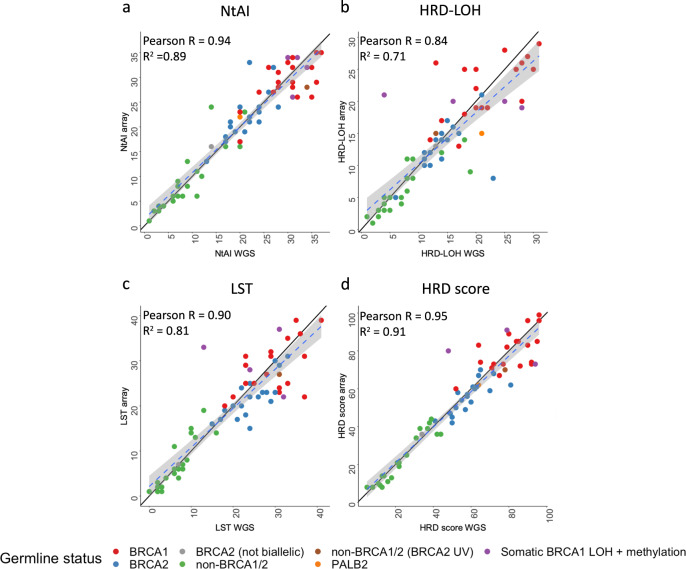
Correlation between the array- and WGS-derived HRD score and HRD score components. WGS data is on the *X*-axis and array data is on the *Y*-axis. **a** array versus WGS-derived NtAI. **b** array versus WGS-derived HRD-LOH. **c** array versus WGS-derived LST. **d** array versus WGS-derived HRD score. Results are for 67 samples and cases are labelled according to their germline BRCA status obtained from Nones et al.^12^. Note: grey shows the *BRCA2* tumour which did not exhibit biallelic inactivation of the gene; purple denotes four non-*BRCA1/2* tumours that had a pattern of somatic mutations reflecting BRCA1 deficiency (three of which had somatic *BRCA1* LOH plus *BRCA1* methylation); brown denotes a non-*BRCA1/2* tumour from a patient carrying a *BRCA2* unclassified variant (UV) plus LOH but with other mutational signatures that suggest HR deficiency; and orange denotes a non-*BRCA1/2* case from a patient with biallelic inactivation of *PALB2* as previously presented^12^. Blue dashed line represents fitted regression line and grey area represents the 95% confidence interval for the fitted regression line. The line of equality (where array is equal to WGS data) is represented by a solid black line (for reference). The scatterplots demonstrate a strong linear relationship between WGS- and array-derived HRD score and HRD score components, as evidenced by the high Pearson’s correlation coefficient and *R*^2^.

Overall, we observed a high correlation between array- and WGS-derived HRD score and HRD score components (Fig. 1, Supplementary Table 1). The Pearson’s correlation coefficient (PCC) for the NtAI, HRD-LOH, LST and HRD score was 0.94, 0.84, 0.90 and 0.95, respectively (Fig. 1). When performing linear regression between the array- and WGS-derived data, we observed *R*^2^ values ranging from 0.71 to 0.91, with relatively small standard errors. (Supplementary Table 1). Taken together, these results suggest a strong linear association between array- and WGS-derived data.

**Fig. 2:**
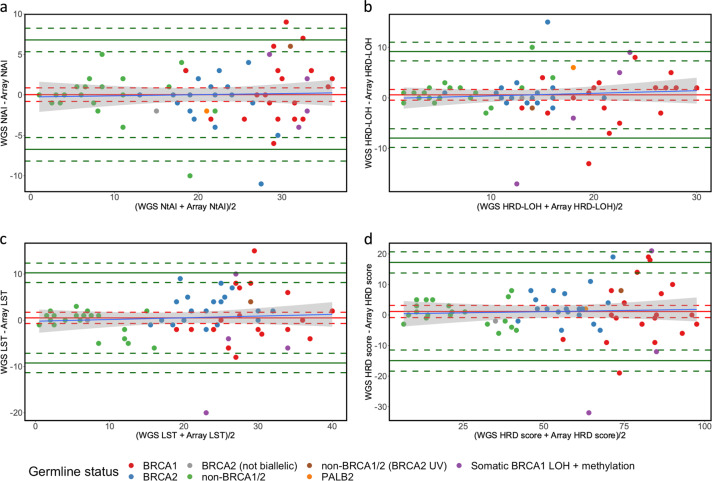
There is a good agreement between the WGS- and array-derived HRD score and HRD score components. Bland-Altman plot for the WGS- and array-derived NtAI (**a**), HRD-LOH (**b**), LST (**c**) and HRD score (**d**) for 67 samples. *X*-axis represents the mean of the two measurements (from WGS and array data) and the *Y*-axis represents the difference between the paired measurements. Solid red line represents mean bias, red dashed lines represent the 95% confidence intervals (CI) of the mean bias. Solid green lines represent the upper and lower limits of agreement and green dashed line represent the 95% CI of the upper and lower limits of agreement. Blue line represents fitted regression line between mean bias and the magnitude of measurements and grey area represents the 95% CI for the fitted regression line. Tumours are colour coded according to their germline BRCA status (see legend to Fig. 1).

As correlation only measures the linear association between two variables (which does not necessarily imply that two methods have good agreement), we complemented the correlation results using a Bland-Altman analysis^16^ (Fig. 2), which measures the extent of agreement between two methods of measurements^17^. The Bland-Altman analysis involves defining limits of agreement between two measurements, based on the mean and standard deviation of the differences between these two measurements. This analysis also provides us with the average difference between the two measurements (WGS and array), termed mean bias.

In the Bland-Altman analysis, 95.52% of the samples (64/67) were within the limits of agreement for the NtAI, HRD-LOH and HRD score. For the LST, we noted 97.01% samples within the limits of agreement (65/67) (Fig. 2). There was a tendency for the variability of the mean bias between array- and WGS-derived parameters to increase as the mean of the array- and WGS-derived parameters increased (Fig. 2). These observations suggest that as the amount of genomic instability increases (characterised by increasing HRD-LOH, LST, NtAI and HRD score), the difference observed between array- and WGS-derived LST and HRD score becomes more variable. Overall, the mean bias observed was small and non-significant across all parameters. This is because the confidence interval of the mean bias included zero (line of equality) within its interval. We noted a mean bias ranging from 0.03 for the NTAI to 1.15 for the HRD score (Supplementary Table 1). Given the absence of significant bias across all the parameters, these results suggest a good agreement between the data derived from array and WGS.

We then investigated how the contribution of HRD score components varied between platforms and HR status (HR deficient v/s HR proficient samples). In both array and WGS, we noted that the NtAI (mean contribution: 38.92%, 95% confidence interval (CI) (36.75%−41.08%) for array; 37.81%, 95% CI (35.47%−40.14%) for WGS) and LST (34.38%, 95% CI (32.04%−36.73%) for array; 34.79%, 95% CI (32.46%−37.11%) for WGS) were the largest contributors to the HRD score, and the HRD-LOH was the smallest contributor (26.70%, 95% CI (24.70%−28.71%) for array; 27.41%, 95% CI (25.27%−29.54%) for WGS).

We observed no significant differences (Wilcoxon signed-rank test, 2-sided *p*-value = 0.80, 0.99 and 0.21 for the HRD-LOH, LST and NtAI, respectively) when comparing the contribution of the different HRD score components between array and WGS (Supplementary Fig. 1). However, we did noticed that when considering the HR status of the sample (using array-derived HRD score) there was an increased contribution of the LST (Wilcoxon signed-rank test, two-sided *p*-value = 0.0096 (array) and 0.007 (WGS)) in the HR deficient samples when compared to the HR proficient ones, and vice versa for the HRD-LOH (Wilcoxon signed-rank test, two-sided *p*-value = 0.0022 (array) and 0.0094 (WGS)), while the NtAI showed no significant differences between the HR status categories (Wilcoxon signed-rank test, two-sided *p*-value = 0.2705 (array) and 0.6217 (WGS)) (Supplementary Fig. 1). This pattern was consistent across both platforms. Similarly, when comparing the distribution of data generated from array and WGS (Kolmogorov–Smirnov test) we noted no significant differences in the NtAI (*p* = 0.99, two-sided *p-*value), LST (*p* = 0.99, two-sided *p-*value), HRD-LOH (*p* = 0.99, two-sided *p-*value) and HRD score (*p* = 0.95, two-sided *p-*value). With respect to the variance in the HRD score components, we noted that the LST had the highest variance (115.81 for array; 124.23 for WGS), followed by the NtAI (99.21; 101.93) and the HRD-LOH (57.13; 62.78), a pattern consistent across both platforms (array and WGS).

To compare the reliability between the WGS- and array-derived data, we computed the intraclass correlation coefficient (ICC), which provides an indication of the consistency of measurements derived from multiple raters (methods). The ICC ranges from 0 to 1, with values between 0.75 and 0.90, and >0.90 indicative of good and excellent reliability, respectively^18^. We observed an ICC of 0.94, 0.84, 0.90 and 0.95 for the NtAI, HRD-LOH, LST and HRD score, respectively (Supplementary Table 1). Taken together, these results indicate a good consistency between WGS- and array-derived scores.

The HRD score is mainly used as a dichotomous method to categorise samples as either HR deficient (HRD score ≥42) or HR proficient (HRD score <42)^1^, therefore it is also important to assess the degree of agreement in HR status classification between the array- and WGS-derived HRD scores. To this end, Fleiss’ kappa^19^ was used as a statistical measure to assess the reliability of the array- and WGS-derived HRD score in performing HR status classification. Fleiss’ Kappa ranges from −1 to 1; a value of <0 is indicative of poor agreement between methods, while a value between 0.80 and 1.00 is indicative of good agreement. When comparing the array to the WGS-derived scores, we observed a 92.54% (62/67 samples) agreement in HR status classification between the array- and WGS-derived data (Fleiss kappa value = 0.83; two-sided *P* value = 1.17E^−11^). While the majority of tumours were consistently classified, five samples (7.46%) were differentially classified by the two methods. Three of these samples were scored as HR deficient by array and as HR proficient by WGS (array HRD scores/WGS HRD scores: 43/41, 42/38 and 44/39), while two were scored as HR proficient by array and as HR deficient by WGS (array HRD scores/WGS HRD scores: 36/44, 36/42); Supplementary Tables 2 and 3). These cases highlight the limitation of using a single genomic parameter to classify the tumours as HR deficient or proficient.

**Fig. 3:**
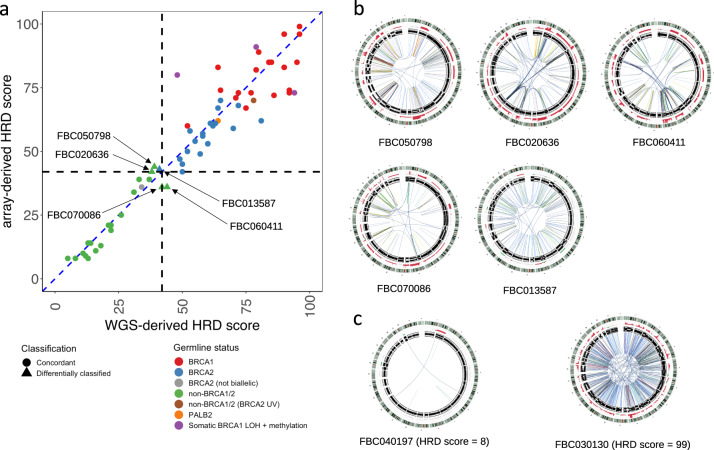
Comparison between HRD scores derived from arrays and WGS, and other genomic features. **a** Scatterplot of array**-** (*Y*-axis) and WGS- (*X*-axis) derived HRD scores; the dashed black line indicates the HRD score threshold of 42, the line of equality (where array is equal to WGS data) is represented by a blue dashed line (for reference) and cases are labelled according to their germline BRCA status obtained from Nones et al.^12^ (see legend to Fig. 1). The five cases differentially classified are labelled and marked with triangles. **b** Circos plots of tumours FBC050798, FBC020636, FBC060411, FBC070086 and FBC013587, those where array and WGS-derived HRD scores disagreed when considering the threshold of 42 for HR status. The circos plots display copy number and B-allele frequency (outer rings) and somatic structural variants (SVs) are represented by lines in the inner ring. Colour of the lines represent SV types. **c** Circos plots for representative tumours classified definitively as HR proficient (HRD = 8) or deficient (HRD = 99), for reference.

The differentially classified samples included four non-*BRCA1/2* tumours (FBC050798, FBC020636, FBC060411 and FBC070086) and one tumour from pathogenic *BRCA2* mutation carrier (FBC013587) (Fig. 3). The genomic architecture of these five tumours is shown as circos plots (Fig. 3b) and copy number profiles (Supplementary Fig. 2) and can be compared to tumours with low and high HRD scores (Fig. 3c). Overall, the copy number profile observed between the platforms were similar, apart from the expected differences in ability to detect high-level copy number amplifications, due to differences in dynamic range. Case FBC013587 (HRD = 43 for array and 41 for WGS, Supplementary Table 2), harboured biallelic inactivation of *BRCA2* and exhibited several genomic features suggestive of HR deficiency, such as a high number of small deletions (>3 bp), with microhomology at the junction of the deletions, prominent mutational signatures^12^ typical of *BRCA1/2* deficient tumours^7,20^ and hence an overall HRDetect score indicative of HR deficiency.

To investigate potential reasons underlying the differential classification of these samples between array and WGS data, we analysed various variables and compared those between samples which were consistently classified to those that were differentially classified. The variables we considered included measures of Log2 ratio signal noise, such as the derivative Log Ratio Spread (dLRS), which consists of the standard deviation of Log2 ratios for the array and WGS data (tumour samples) and the Median of the Absolute values of all Pairwise Differences (MAPD) for the array and WGS data (tumour samples). Other variables we considered included: deviation in the tumour segmented Log2 ratio data between WGS and array; the correlation between the tumour Log2 ratio data derived from array and WGS; tumour sample purity (ACF) and sequencing coverage for the normal and tumour data. We did not find evidence that these variables could explain the discrepancies in HR classification for the five samples (Supplementary Fig. 3).

We further generated a statistical model to investigate how various specific quality metrics affect the relationship between the array- and WGS-derived HRD scores. The model initially included eight variables in the regression: purity of the tumour sample (ACF), dLRS for the tumour (array and WGS), tumour coverage and normal coverage, correlation in the tumour Log2 ratio data space, the deviation in the tumour segmented Log2 ratio and the WGS-derived HRD score. Overall, these analyses suggest that more often than not, the variance in WGS-derived HRD score alone, is sufficient to account for the variance in array-derived HRD score. Further details of these analyses are found in the supplementary material (Supplementary Note, Supplementary Figs. 4 and 5).

### Comparing original and downsampled WGS-derived HRD scores

We then determined if low coverage WGS data could be used to derive HRD scores and HR classification status consistent with the original tumour coverage (~70×) WGS data. We performed an incremental downsampling of normal and tumour original WGS data on the cohort of 67 familial breast cancers to reach an average coverage of approximately 30X, 15X and 10X for both tumour and normal WGS data (Supplementary Table 2). During the downsampling process, ascatNgs failed to run on three samples for both the 15X and 10X downsampling. Therefore, the results presented below include 64 samples, where ascatNgs successfully generated data for all downsampling tiers (30X, 15X and 10X). We discuss potential reasons why these three samples failed ascatNgs copy number estimation in Supplementary Fig. 6.

We observed a high correlation between the original and the downsampled WGS-derived HRD score and HRD score components (Pearson’s correlation coefficient between 0.79 and 0.98; Supplementary Table 4). Performing linear regression for the HRD score components between the original and downsampled WGS data (Supplementary Table 4), we noted small standard errors and relatively high *R*^2^ values (ranging from 0.62 to 0.96). Taken together, these results indicate that there is a strong linear relationship between the original and downsampled WGS data. However, we noticed that the HRD-LOH component deteriorated rapidly with downsampling, in comparison to other HRD score parameters (*R*^2^ of 0.90 at 30×, 0.63 at 15X and 0.62 at 10×), suggesting a reduced capability at calling HRD-LOH in lower coverage WGS. A Bland-Altman analysis revealed a relatively small mean bias between the original and downsampled WGS-derived HRD (ranging from −0.75 to 4.77), which is indicative of a good agreement between original and downsampled WGS-derived data. The results of these analyses are summarised in Supplementary Table 4.

As before, we complemented our Bland-Altman analysis of agreement by assessing the intraclass correlation coefficient and the Fleiss Kappa. A Fleiss Kappa value of 0.93 (two-sided *P* value < 2E10^−16^) was obtained and the intraclass correlation coefficient calculated ranged from 0.85 to 0.96 (Supplementary Table 4). These results are indicative of good consistency between the original and downsampled WGS data. We also noted an excellent agreement between the original and downsampled WGS-derived HR classification status: 93.75% (60/64) of tumours were consistently classified for all coverages (original coverage, 30X, 15X and 10X), with a misclassification rate of 6.25% (4/64) when the WGS data was downsampled to 30X, 15X and 10X (Supplementary Tables 2 and 5).

Four samples (FBC013587, FBC020636, FBC050798 and FBC050558) exhibited inconsistent classification between the original WGS and downsampled WGS data, three were previously differently classified when comparing their array- to WGS-derived scores (FBC013587, FBC020636 and FBC050798; Supplementary Table 2). The original WGS-derived HRD scores for these tumours (FBC013587:41, FBC020636:38, FBC050798:39 and FBC050558:37) were close to the threshold of 42, and in most cases the downsampling led to an increased HRD score. In fact, for 48/64 cases, we observed an increased HRD score compared to the original score, across at least two downsampling tiers. This could arise through the downsampling process, as the noise in allele frequency estimation increases in the lower coverage data. To understand possible reasons underlying the non-congruent HR classification for these samples, we looked at some of the factors that could influence HRD score derivation. The first factor we considered was tumour cellularity (ACF). The cellularity of these four tumours were relatively high (69.40–73.53%), except for sample FBC020636 which had a cellularity of 51.25% (Supplementary Table 2).

Regions of high and low G + C bases are challenging to sequence, resulting in poor coverage; a phenomenon known as GC bias. Hence, we derived various GC bias metrics to ascertain if they could provide insight into why these four samples were not congruously classified (Supplementary Fig. 7). We did not notice a tendency for the four non-congruent samples to have worst GC bias compared to the congruently classified samples. We also derived WGS performance metrics and did not notice any difference between the congruent and non-congruent samples for these. However, because the downsampling performed was random, it is possible that this process resulted in a non-uniform genome coverage, which may have impacted the ability to determine scores and ultimately affected our ability to accurately calculate the HRD score. Also, because a non-uniform genome coverage may artifactually result in an increased number of CN breakpoints, this could ultimately increase the HRD score calculated; this could therefore explain the tendency for downsampled WGS-derived HRD score to be higher than its original counterpart, as observed in the Bland-Altman analysis (Supplementary Table 4).

## Discussion

Fundamental to calculating the HRD score is the robust determination of copy number (CN) data, which can be obtained using either SNP arrays, WES or WGS. Studies comparing WES- to WGS-derived CN data have shown that WGS provides a much more homogenous distribution of quality parameters (genotype quality, coverage depth and minor read ratio) compared to WES^21,22^. The challenges with using WES to determine CNs relate to the non-adjoining nature of the captured exons and CNs extending outside the capture regions^23^. Indeed, Zare et al.^24^ demonstrated CN detection tools using WES data have relatively limited performance, due to the presence of additional biases that arise as part of the hybridisation process and uneven read distribution in exonic regions^25^. Nevertheless, WES has been shown to reflect copy number data and HRD scores to that obtained from array data^1,8,26^.

In this study, we demonstrated with various statistical analyses (Pearson’s correlation, Bland-Altman, interclass correlation coefficient and Fleiss’ Kappa) that HRD scores and individual parameters of the HRD score derived from WGS closely reflect those obtained by SNP array. Similarly, compared to Sztupinszki et al’s^8^ findings comparing HRD scores derived from array and WES^8^, we report a good correlation between the array and WGS-based HRD score. However, in comparison to Sztupinszki et al’s^8^ findings, we report a higher Pearson’s correlation between scores estimated from arrays and WGS (HRD score: 0.95; NtAI: 0.94; HRD-LOH: 0.84; LST: 0.90) when compared to the correlation they reported for scores estimated from WES (HRD score: 0.87; NtAI: 0.84; HRD-LOH: 0.73; LST: 0.79)^8^ Also, while Sztupinszki et al. reported that the estimation of the HRD-LOH was lower in the WES- compared to array-estimated data, for the WGS data, we reported no differences across all the parameters, including the HRD-LOH, both in terms of their contribution, but also in term of their distribution between both platforms. The higher Pearson’s correlation coefficient achieved using WGS over WES might be expected, given the aforementioned advantages of WGS over WES for calling CNVs^21–25,27^. This demonstrable utility of WGS for scoring HRD emphasises the powerful nature of WGS technology over arrays and WES, given WGS yields a more detailed characterisation of the patient’s complete cancer genome, including single-nucleotide variants (SNVs), structural variants and collective mutational signatures, which in combination with the HRD score, has better discriminating powers for predicting HR status in tumours, such as used by HRDetect^7,12^.

Our analyses also establish that HRD scoring can be achieved from lower coverage WGS data, based on our results from downsampled genomes. We demonstrate a high correlation, a good agreement, as well as good HR classification congruency between the original and downsampled WGS. However, when considering lower coverage genomes, the sensitivity to fully characterise somatic mutations (e.g. SNVs, breakpoints and copy number changes) becomes compromised, especially in tumours of low cellularity or when sequencing data presents strong GC bias. The cost savings therefore achieved through low coverage sequencing has to be balanced with the sensitivity for robustly calling somatic mutations.

The discrepancy observed here between HRD status classification in a minority of tumours between either the array and WGS comparison or between the original and downsampled WGS comparison raises some concern with using only HRD scores for therapy decision making (i.e. whether to give platinum-based chemotherapy or PARPi or not). We found that several tumours with HRD scores close to the threshold of 42 switched between being proficient or deficient in these comparisons, whereas with the implementation of HRDetect^7^, that uses multiple lines of evidence for the stratification of these tumours as proficient or deficient, presented a more distinct separation^12^. This highlights the value of utilising multiple sources of genomic information, where possible, to inform on the HR status of a tumour. This might include germline/somatic mutation status of key HR genes (e.g. *BRCA1, BRCA2, PALB2*), whether the second allele is affected by somatic mutation or methylation, and critically, considering other genomic patterns such as mutational signatures^7,12^. All of these genomic parameters can be obtained through WGS, and so this technology would be ideal for clinical service delivery, if available. Array- and WES-derived genomic assessment of tumours appear useful alternatives, with the associated limitations described being important considerations in the treatment decision making process.

## Methods

### Cohort

The cohort and genomic data used herein was previously described in detail^12^. In the current study we analysed a series of 67 patients that had both array and WGS data for normal and tumour samples. The patients harboured known pathogenic germline mutations in *BRCA1* or *BRCA2* or were from individuals in high risk, breast cancer families who were negative for *BRCA1* or *BRCA2* mutations (non-*BRCA1/2*) following germline testing. Each tissue bank providing samples had received written informed consent from all the patients involved. Work performed was covered by Human Research Ethics Committee approval from the University of Queensland (2005000785) and QIMR Berghofer Human Research Ethics Committee (P3527).

### SNP array and whole-genome sequencing data

The germline and tumour DNA were tested using Illumina Infinium arrays (Illumina, San Diego, CA, USA) according to manufacturer’s instructions. DNA from tumour and matched normal underwent whole-genome paired-end sequencing using an Illumina X-Ten. Sequence reads were trimmed using Cutadapt (version 1.11)^28^ and aligned to GRCh37 using BWA-MEM (version 0.7.12)^29^. Duplicate alignments were marked with Picard (version 1.129, http://picard.sourceforge.net) and BAM files were coordinated-sorted using Samtools (version 1.3)^30^. Mean coverage was determined using samtools and the following command: samtools depth -a **bam_file** | awk ‘{sum + =$3} END { print “Average = “,sum/NR}’ > **output_coverage**. Tumours were sequenced to an average read depth of ~70 × (range of 55X–104X) and normal to ~36 × (range of 28X–44X). The WGS data have been deposited in the European Genome-phenome Archive (EGA) repository under the accession code EGAS0000100330^31^.

### Derivation of B-allele frequencies, logR ratios, HRD score and HRD score components from array- and WGS-derived data

SNP arrays were scanned on an iScan (Illumina) and data was processed using the Genotyping module (v1.9.4) in GenomeStudio v2011.1 (Illumina, San Diego CA). Genomestudio calculates B-allele frequencies (BAF) and LogR ratios (LRR) normalised against clusters file (reference data generated from a large set of individuals provided by the manufacturer). BAF and LRR data was used as an input to ASCAT copy number R package (https://github.com/Crick-CancerGenomics/ascat)^13^, which is an algorithm inferring a sample’s allele-specific copy number profile. We used 1,855,219 SNP positions in the arrays and 1,855,235 positions in the WGS data to derive the copy number data. This data was then used as an input to the script provided in the supplementary methods of Marquard et al.^15^ to derive the score for the individual components (HRD-LOH, LST and NtAI) and the overall HRD score.ascatNgs^14^ was used to generate allele-specific copy number profile for the WGS data using the paired tumour/normal BAM files as input. This data was then used to calculate the HRD score as described above.

### Downsampling of WGS data

Downsampling of the original normal and tumour BAM files was performed using the samtools (version 1.3)^30^ library function: samtools view -h -s **x**, where **x** represents the desired percentage of downsampling required (we used values that would result in an approximate coverage of 30X, 15X and 10X, depending on the coverage of the original data). After downsampling, coverage of the downsampled data was calculated and the downsampled BAM files were indexed using the library function samtools index. Picard (http://broadinstitute.github.io/picard/) was used to generate WGS performance metrics (CollectWgsMetrics) and GC bias metrics (CollectGcBiasMetrics). Subsequently, paired tumour/normal downsampled and indexed BAM files were used as input to ascatNgs that generated allele-specific copy number profile for the paired tumour/normal BAM files. This data was then used to calculate the HRD score, as described above.

### Derivation of dLRS, MAPD

The dLRS for array and WGS data was derived using an adaptation of the dLRS function from the ADM3 version 1.3 package in R (https://cran.r-project.org/src/contrib/Archive/ADM3/). The dLRS function was applied on the ASCAT- and ascatNGS-derived Log2 ratio data. The MAPD for the array and WGS tumour data was derived by computing the median value of all absolute difference in Log2 ratio between genomically adjacent probes.

### Derivation of deviation in segmented Log2 ratio and correlation in Log2 ratio data

Because the ~1.8-M SNP positions used to derive the copy number were not exactly the same for the array- and WGS- copy number data, we created 288,113 bins of 10 kb each, representative of chromosome 1–22. For each bin, we computed the median of the segmented Log2 ratios mapping to this region and mapped this value to its matching 10 kb bin. We then calculated the deviation in segmented Log2 ratios by taking the median of absolute differences in Log2 ratios, across all bins between the array and WGS for the tumour samples.

To derive the correlation in Log2 ratio data, we applied a similar approach, but instead of segmented Log2, we used the unsegmented data. For each 10 kb bin, we computed the median of the Log2 ratios mapping to this particular region and mapped this value to its matching 10 kb bin. As a measure of correlation between array and WGS-derived Log2 ratios we used the Spearman correlation coefficient.

### Measurement of agreement between array-, original-WGS- and downsampled-WGS-derived HRD score and HRD score components

The R package BlandAltmanLeh (https://cran.r-project.org/web/packages/BlandAltmanLeh/vignettes/Intro.html) was used to determine the Bland-Altman parameters required to generate the Bland-Altman plots, including the mean bias and its 95% confidence intervals, the upper and lower limits of agreement, together with their 95% confidence intervals.

To assess the agreement between array, original and downsampled WGS-derived HR classification, we used Fleiss’ kappa and the intraclass correlation coefficient (ICC3). The Fleiss kappa was computed using the irr package (https://cran.r-project.org/web/packages/irr/index.html) and the ICC3 was computed using the DescTools package (https://cran.r-project.org/web/packages/DescTools/index.html).

### Statistical analyses

All analyses were done in the R statistical environment^32^.

### Reporting summary

Further information on experimental design is available in the Nature Research Reporting Summary linked to this paper.

## Supplementary information

Supplementary Material

Reporting Summary Checklist

## Data Availability

All the data supporting the findings of this study are publicly available in the figshare repository. Segmentation data from ASCAT (array data) are available from 10.6084/m9.figshare.9808496 and ascatNGS (for original (10.6084/m9.figshare.9808505) and downsampled WGS data (30×: 10.6084/m9.figshare.9808511, 15×: 10.6084/m9.figshare.9808514, 10×: 10.6084/m9.figshare.9808517)). Supplementary Figure 1 was generated using these data. The HRD score and HRD score components matrix obtained for the array data, are available from the following record: 10.6084/m9.figshare.9808526. Similar data for the original coverageWGS data are located here: 10.6084/m9.figshare.9808529 and the HRD score matrices for the downsampled WGS data are found here: 10.6084/m9.figshare.9809978 (30×), here: 10.6084/m9.figshare.9810053 (15×) and here: 10.6084/m9.figshare.9820646 (10×). Figures 1, 2 and 3 were derived from these data. The WGS performance and GC bias metrics for the four differentially classified samples between original coverage and downsampled WGS-derived HRD score are available here: 10.6084/m9.figshare.9810164 (WGS performance metrics) and here: 10.6084/m9.figshare.9810212 (GC bias metrics). Supplementary Figure 3 is derived from these data. The WGS performance and GC bias metrics for the three samples that failed to run using downsampled WGS data are available here: 10.6084/m9.figshare.9810218 (WGS performance metrics) and here: 10.6084/m9.figshare.9810221 (GC bias metrics). Supplementary Figure 6 is derived from these data. Data for the Circos plots of tumours FBC050798, FBC020636, FBC060411, FBC070086, FBC013587, FBC040197 and FBC030130, are also available on figshare in the following data records: 10.6084/m9.figshare.12271529 (FBC013587), 10.6084/m9.figshare.12271868 (FBC040197), 10.6084/m9.figshare.12271961 (FBC050798), 10.6084/m9.figshare.12271859 (FBC070086), 10.6084/m9.figshare.12271538 (FBC060411), 10.6084/m9.figshare.12271871 (FBC020636) and 10.6084/m9.figshare.12271877 (FBC030130). Supplementary tables 1–5 are available in figshare as part of this data record 10.6084/m9.figshare.12301898^33^. The whole-genome sequencing data analysed during this study are available in the European Genome-phenome Archive here: https://identifiers.org/ega.study:EGAS00001003305.

## Code availability

The code used to analyse the data and generate figures presented in this publication is available upon request.
