# Supplementary material for: Using whole-genome sequencing data to derive the homologous recombination deficiency scores
Source: NPJ Breast Cancer. 2020 Aug 7;6:33. doi: 10.1038/s41523-020-0172-0 (PMC7414867; doi:10.1038/s41523-020-0172-0)
Supplement: Supplementary file 1 — Supplementary Material [file 41523_2020_172_MOESM1_ESM.pdf]

Using Whole Genome Sequencing data to derive the homologous recombination deficiency scores

Xavier Marc de Luca[1], Felicity Newell[2], Stephen H. Kazakoff[2], Gunter Hartel[2], Amy E. McCart Reed[1], Oliver Holmes[2], Qinying Xu[2], Scott Wood[2], Conrad Leonard[2], John V. Pearson[2], Sunil R. Lakhani[1,3], Nicola Waddell[2], Katia Nones[£2], and Peter T. Simpson[£1*]

[1] Centre for Clinical Research, Faculty of Medicine, The University of Queensland, Brisbane, QLD, Australia

[2] QIMR Berghofer Medical Research Institute, Brisbane, QLD, Australia

[3] Pathology Queensland, Royal Brisbane & Women's Hospital, Brisbane, QLD, Australia

[£] These authors contributed equally to the study.

*Corresponding author: p.simpson@uq.edu.au

Supplementary Materials

Supplementary Figure 1: Analysis of the contribution of the HRD score components between platforms and HR status

Supplementary Figure 2: Whole genome copy number profiles for the five cases with differentially classified HR status

Supplementary Figure 3: Exploration of various factors which could have influenced the differential HR classification between array- and WGS-derived HR status

Supplementary Note: Modelling the effect of various quality metrics on the regression between array- and WGS-derived HRD score

Supplementary Figure 4: Building a more comprehensive model of the array- and WGS-derived HRD score

Supplementary Figure 5: Relating array and WGS signal noise to absolute differences between the array- and WGS-derived HRD score

Supplementary Figure 6: WGS performance and GC bias metrics for the three samples where ascatNgs failed

Supplementary Figure 7: WGS performance and GC bias metrics for the normal and tumour samples

Supplementary Figure 1

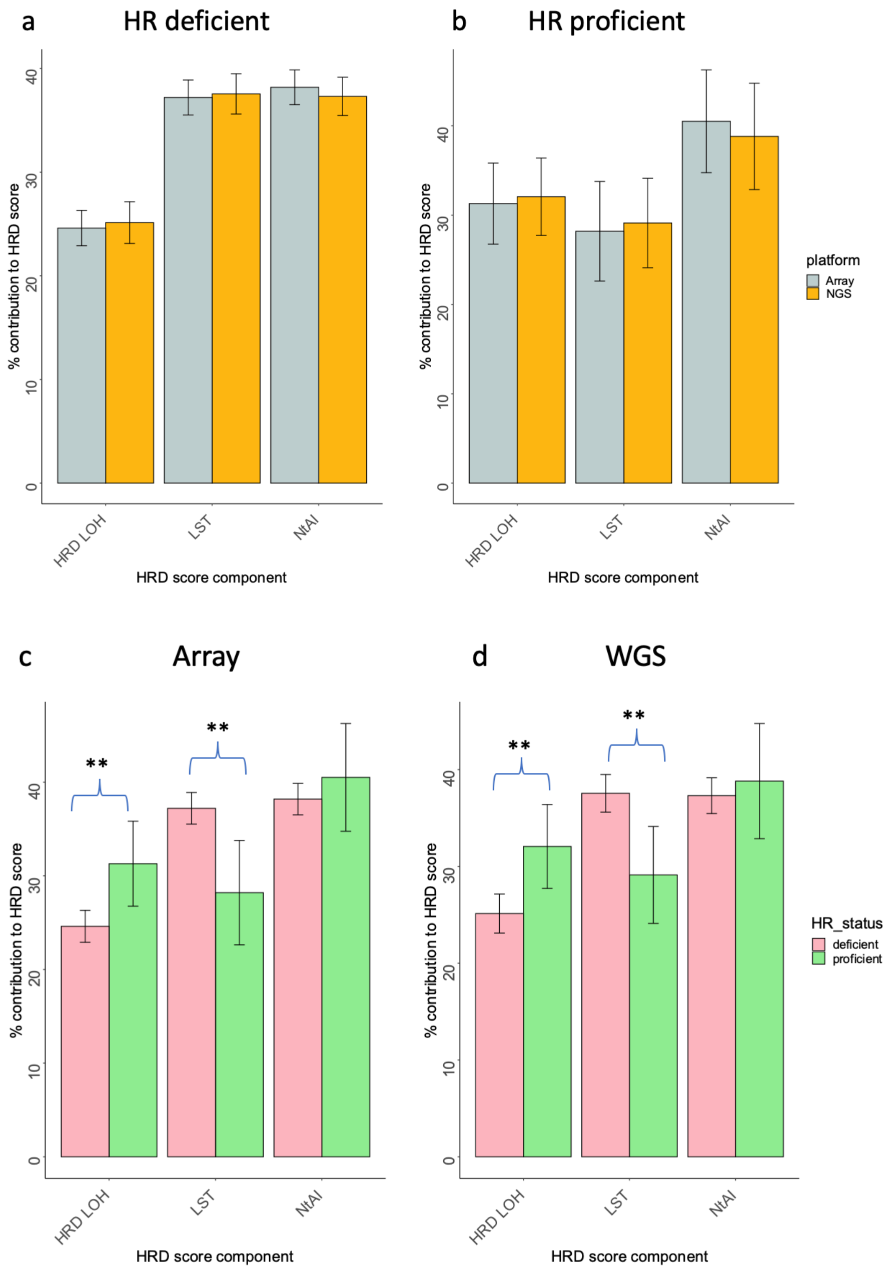

Supplementary Figure 1. Analysis of the contribution of the HRD score components between platforms and HR status. The graph represents the mean component contribution±SEM across HR deficient (**a**) (n = 46), proficient (**b**) (n = 21) samples and across the array- (n = 67) (**c**) and WGS- (n = 67) (**d**) derived data. ** indicates a significance of less than *p* = 0.01. While we observed no significant differences in the contribution of the different HRD score components between array and WGS platforms, the contribution of the LST was greater in the HR deficient compared to HR proficient samples, and *vice versa* for the HRD-LOH.

Supplementary Figure 2

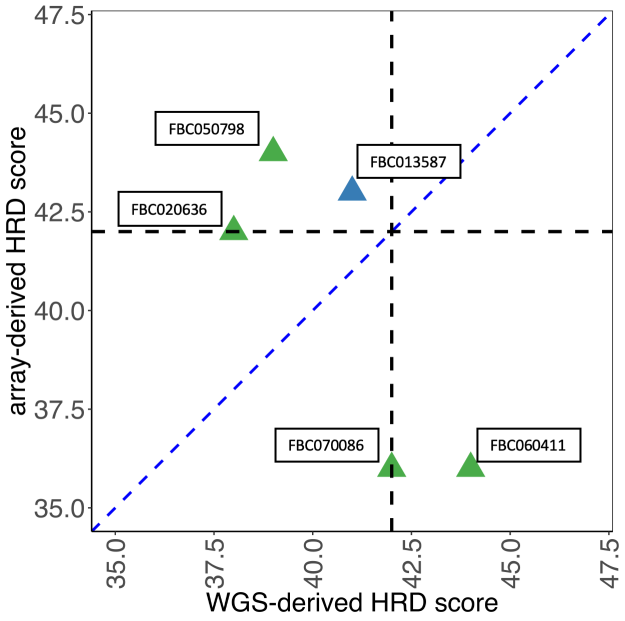

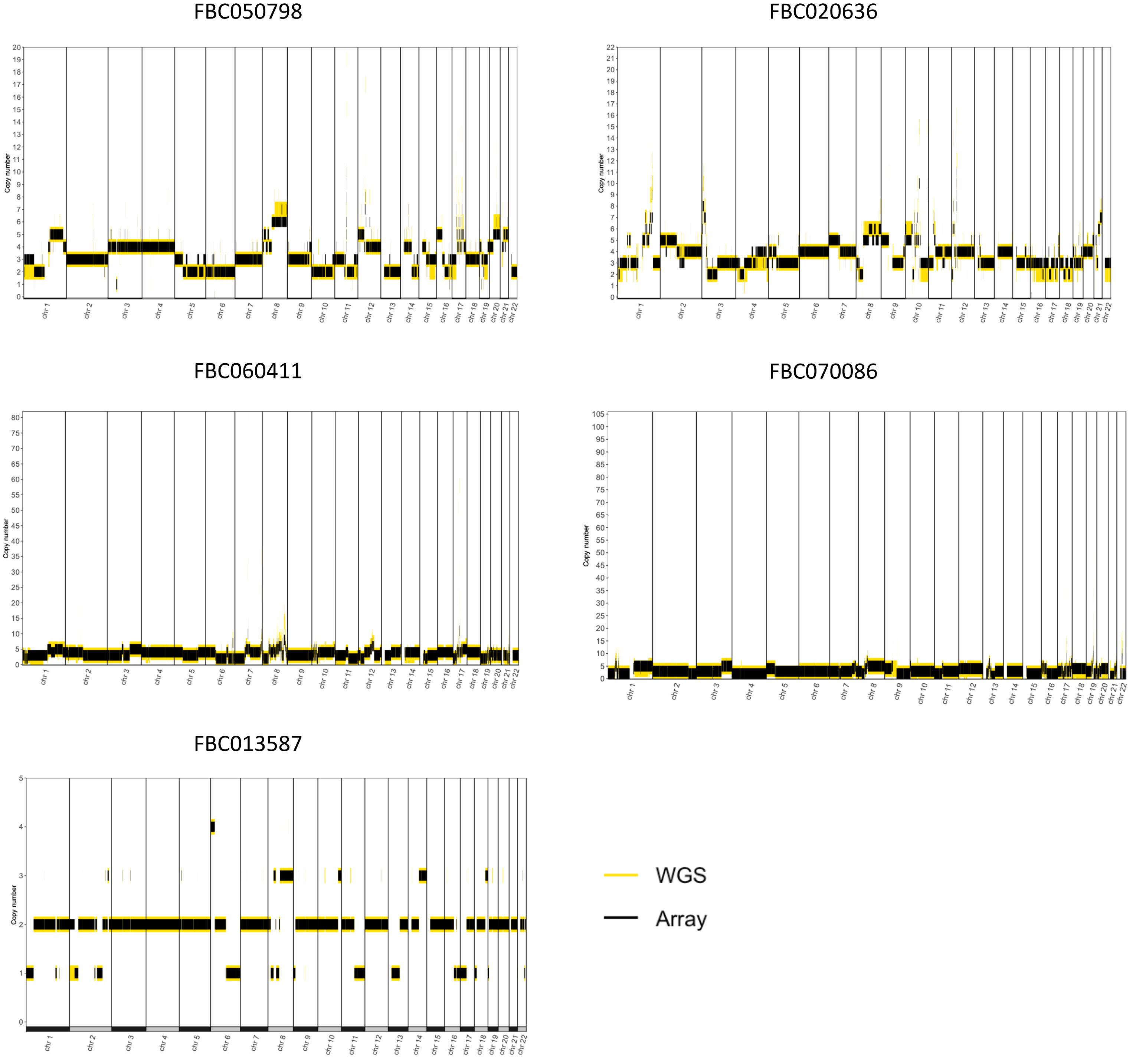

Supplementary Figure 2: Whole genome copy number profiles for the five cases with differentially classified HR status.
Inset is from Figure 3 showing the five samples with differentially classified HR status. The copy number profiles along chromosomes 1-22 are shown with array-derived data (black), overlaid with WGS-derived data (yellow). There was, in general a good match between the copy number transitions between array and WGS data.

Supplementary Figure 3

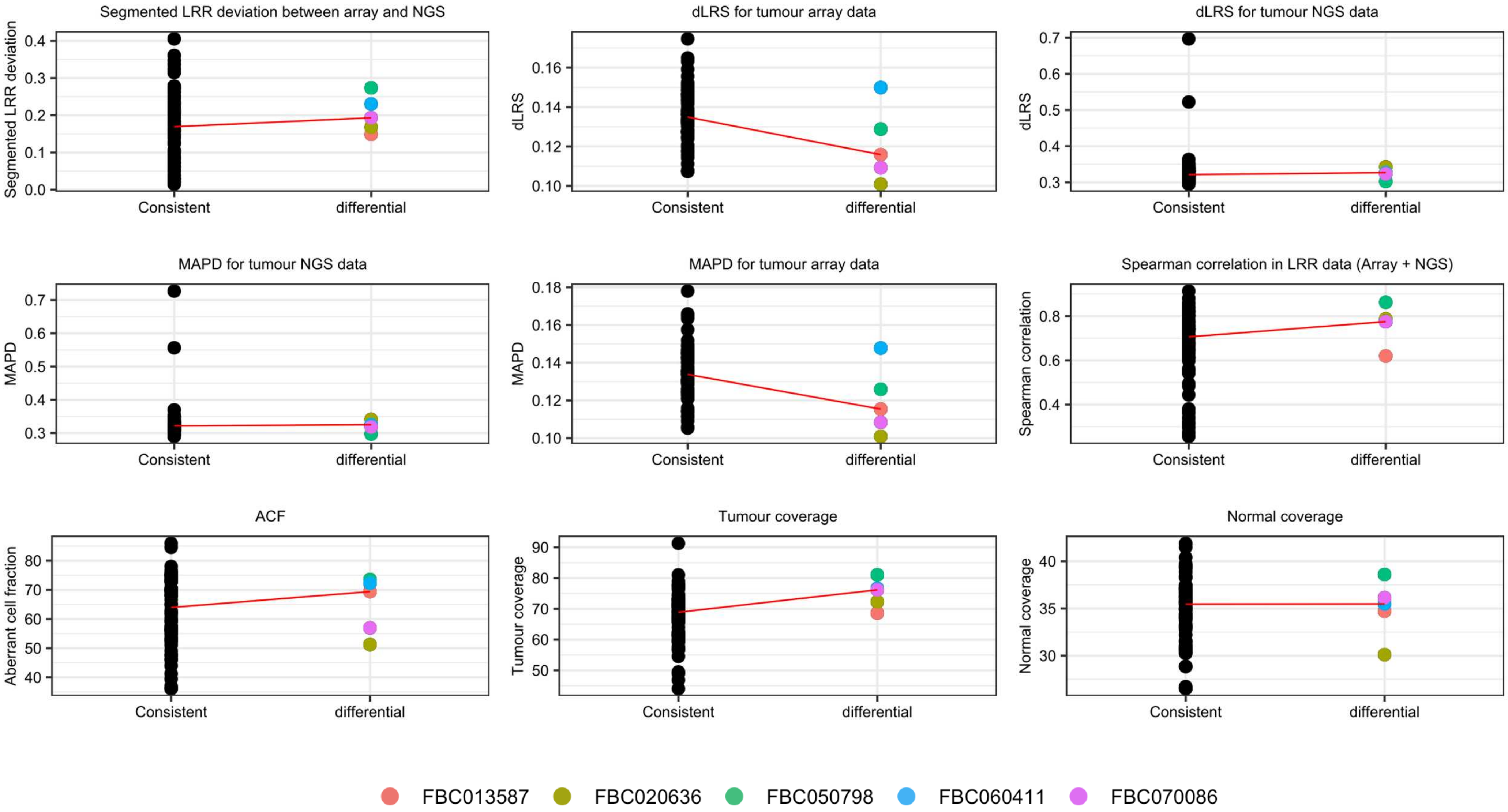

Supplementary Figure 3. Exploration of various factors which could have influenced the differential HR classification between array- and WGS-derived HR status. The five samples which were differentially classified between array and WGS-derived HRD score are individually colour-coded. Red line represents the median between the group of samples which were consistently classified between array and WGS and those that weren't. The parameters plotted represent the tumour data, except for the normal coverage data. The plots do not clearly reveal a particular variable which is worst in the differentially classified samples.

Supplementary Note

Modelling the effect of various quality metrics on the regression between array- and WGS-derived HRD score

To understand how the various quality metrics we derived may affect the relationship between the array- and WGS-derived HRD score, we wanted to generate a statistical model which would include these as variables. Given that the MAPD and dLRS are both proxies for the level of noise in the array and WGS data, and that they had a very high Pearson's correlation (0.99), we decided to keep the dLRS and exclude the MAPD from our initial model to avoid multicollinearity. As such, we included the following eight variables in the regression: purity of the tumour sample (ACF), dLRS for the tumour (array and WGS), tumour coverage and normal coverage, correlation in the tumour Log_2 ratio data space, the deviation in the tumour segmented Log_2 ratio and the WGS-derived HRD score. To identify important features predictive of the array derived HRD score, yet maintain parsimony, we used the caret package (http://topepo.github.io/caret/index.html) to perform recursive feature elimination (RFE) to select the most relevant variables with 10-fold cross-validation repeated 10 times. The 67 samples were divided into a training (n = 61) and testing set (n = 6) using a balanced split to ensure similar representation of HRD scores.

We allowed the RFE algorithm to select from a minimum of two variables to a maximum of six from our total of eight variables (including the WGS-derived HRD score). Analysis of the results suggested that the inclusion of three variables (WGS-derived HRD score, dLRS of array and WGS tumour samples) were optimal for the regression. Our model was defined as follows (**Supplementary Figure**

4): $\log \text{HRD score}_{Array} = -0.24 + 1.00 * \log \text{HRD score}_{WGS} - 1.98 * \text{Array dLRS}_{Tumour} + 1.45 * \text{WGS dLRS}_{Tumour}$. The final model generated had a Root Mean Square Error (RMSE) of 0.14 and an R^2 of 0.97 and testing the model on the hold-out set of six samples resulted in an RMSE of 0.20. Using the predictorEffects function in the effects package (https://cran.r-project.org/web/packages/effects/index.html), we generated predictor effect plots for our model. These revealed a weak negative trend of the predicted array-derived HRD score with increasing array-derived dLRS but a positive trend with the WGS-derived dLRS (**Supplementary Figure 4**). In both cases, the bands of confidence around the predicted array-derived HRD score values were wide. Unexpectedly, the WGS-derived HRD score had a positive relationship with the predicted array-derived HRD score, together with tight bands of confidence around the predicted values.

To investigate further how the WGS- and array-derived dLRS relates to the difference in WGS- and array-derived HRD score, we categorized the absolute HRD score difference (|Δ HRD score|) between WGS and array into three categories: $0 \geq$ |Δ HRD score| ≤ 5; $6 \geq$ |Δ HRD score| < 10 and $10 \geq$ |Δ HRD score| ≤ 32. Performing the Jonckheere-Terpstra test, we noted a statistically significant (JT statistic = 705, one-sided *p*-value of 0.0401) trend of higher median WGS-derived dLRS with higher categories of absolute HRD score difference. We performed the same analysis for the array-derived dLRS, however noted no significance using the Jonckheere-Terpstra test (Jonckheere-Terpstra statistic = 686.5, one-sided *p*-value = 0.0659). Importantly, it was noted that sample FBC050467 (a non-*BRCA1/2* with somatic LOH of *BRCA1* and a BRCA1-like pattern of mutations) and FBC208006 (a germline *BRCA1* mutant)

had a very large tumour WGS-derived dLRS, together with a large |Δ HRD score| (**Supplementary Figure 5**).

In an attempt to understand some of the factors underlying the noisy WGS signal in samples FBC050467 and FBC208006, we interrogated previously derived quality metrics. The cellularity (FBC208006: 72.70% and FBC050467: 75.00%) and tumour coverage (FBC208006: 84.04X and FBC050467: 57.28X) of these samples did not appear to be problematic. However, further analysis of GC bias and WGS performance metrics for these tumour samples revealed an elevated AT_DROPOUT metric (FBC208006: 12.97% and FBC050467: 13.65%) compared to other samples, indicating that approximately 13% of total reads that should have mapped to regions with a GC content ≤ 50% mapped elsewhere (**Supplementary Figure 5**). Also, sample FBC208006 previously failed to generate data when running ascatNGS on the 10X downsampling BAM file (**Supplementary table 2**). As these two samples were outliers with respect to their AT_DROPOUT statistic, we decided to remove them from the data and repeated the Jonckheere-Terpstra test; we now noted a lack of significance (Jonckheere-Terpstra statistic = 591, one-sided p-value = 0.1442).

In order to provide us with an indication of the proportion of explained variance contributed by the predictors included in the final model, we used the relaimpo package (https://cran.r-project.org/web/packages/relaimpo/) to calculate the proportion of the variance explained by all the variables included in the model, using the lmg approach[1], which enables calculation of the relative contribution of a given predictor to the R^2 of the model (proportion of the variance explained by all the variables included in the model), with consideration of the sequence of predictors

which appear in the model. Performing 1000 bootstraps of this statistic, we noted that the 95%CI for the relative contribution to the explained variance for the WGS-derived HRD score ranged from 0.87 to 0.96, whereas that of the array-derived dLRS ranged between 0.0011 to 0.087 and that of the WGS-derived dLRS ranged between 0.0008 to 0.043.

Overall, these analyses suggest that more often than not, the variance in WGS-derived HRD score alone, is sufficient to account for the array-derived HRD score, however in circumstances where a sample has very noisy WGS signal (e.g. FBC050467 and FBC208006), then some of variation in the WGS-derived HRD score not explaining the array-derived HRD score by itself, could be explained by the higher noise (as proxied by the WGS-derived dLRS) in that sample. Notwithstanding these extreme cases of noisy signal, we observe that the relative contribution of the array- and WGS-derived dLRS to the explained variance of the model remains very small.

References

1 Lindeman, R. H., Merenda, P. F. & Ruth, Z. Gold. 1980. *Introduction to bivariate and multivariate analysis*, L743.

Supplementary Figure 4

a

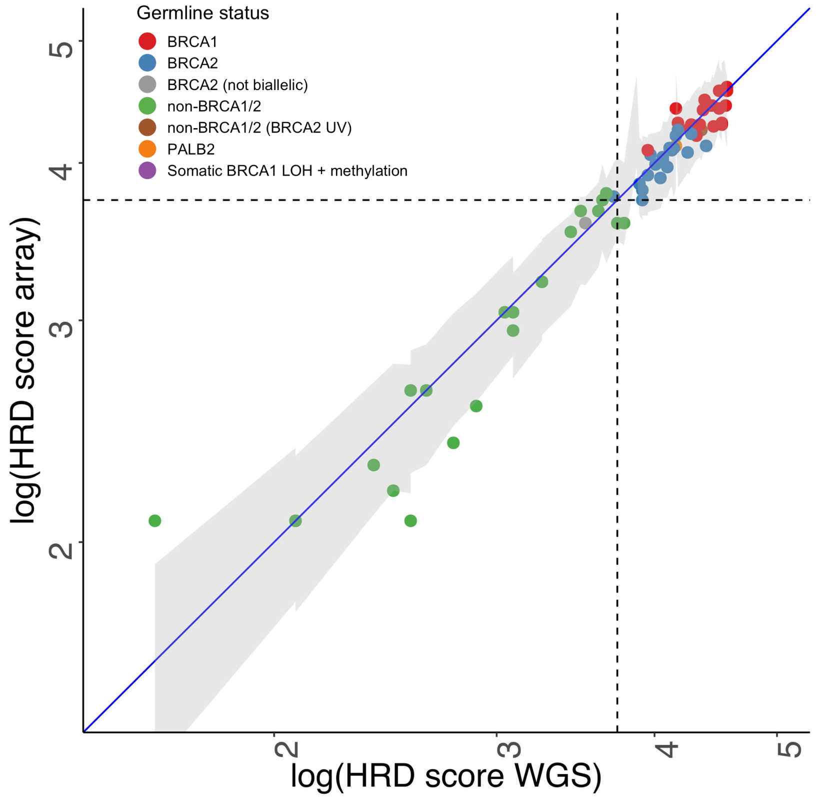

b

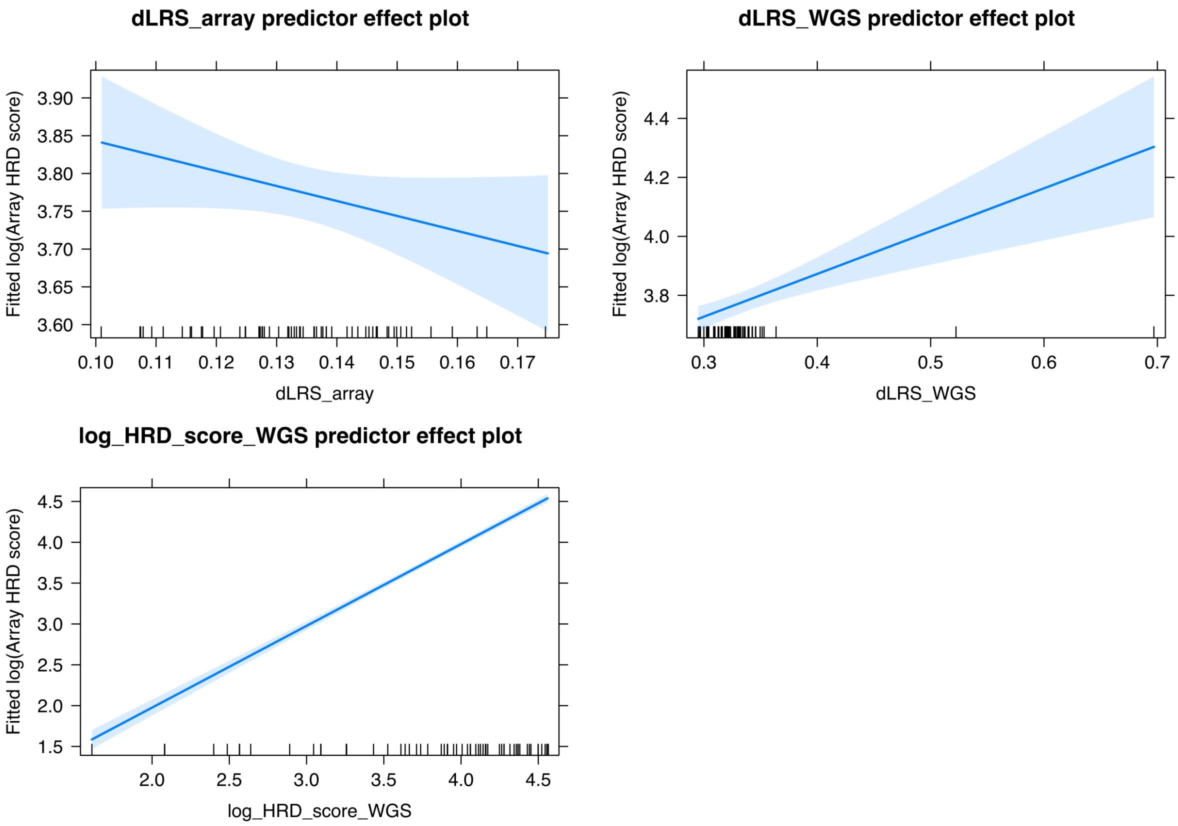

Supplementary Figure 4: Building a more comprehensive model of the array- and WGS-derived HRD score.
(a) Plot of the final linear model of the HRD score derived from array and WGS. Actual HRD score data is represented by the coloured dots, where samples are color-coded according to their germline mutation status. The shaded area represents predicted intervals for each the array-derived HRD score data point (n = 67), based on the fitted model: log(Array HRD score) = −0.24+ 1.00∗log(HRD score_WGS) − 1.98∗Array_dLRS_Tumour + 1.45∗WGS_dLRS_Tumour.The black dashed lines indicate the HRD score threshold of 42 and the blue dashed line indicate the line of equality. **(b)** predictor effect plot of the final model. This plot represents what happens with the predicted array HRD score when a single variable is changed as the other predictors are held fixed. The shaded area is a 95% pointwise confidence band for the fitted values, based on standard errors computed from the covariance matrix of the fitted regression coefficients. The small vertical black bars represent the actual data points. We note a weak trend between the predicted array-derived HRD score and dLRS from array and WGS, with large confidence bands for the predicted values. This trend is negative for the array-derived dLRS but positive for the WGS-derived dLRS. On the other hand, we noted a strong positive trend between the WGS-derived HRD score and predicted array-derived HRD score.

Supplementary Figure 5

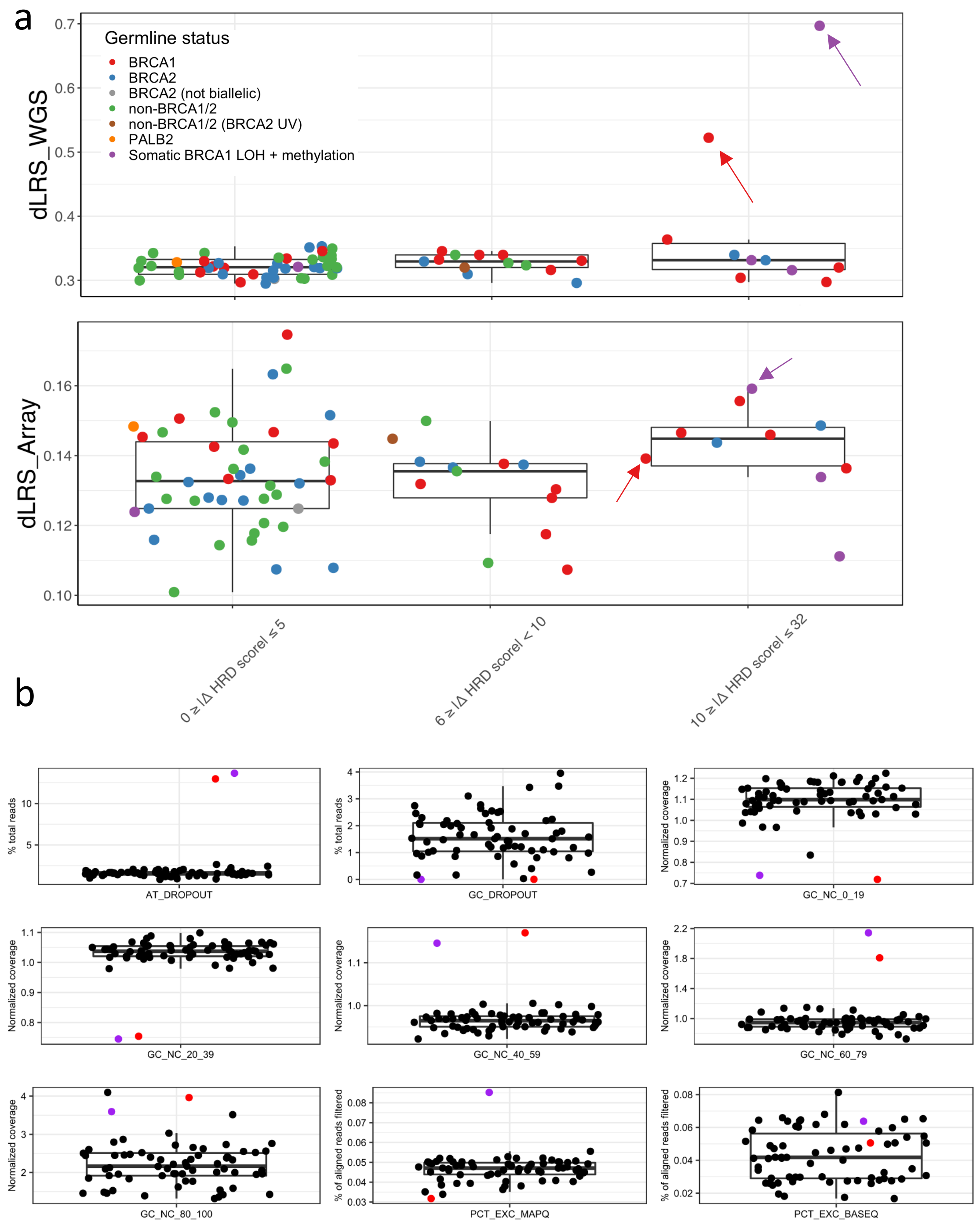

Supplementary Figure 5: Relating array and WGS signal noise to absolute differences between the array- and WGS-derived HRD score. **(a)** Distribution of the WGS- and array- derived dLRS with increasing absolute HRD score difference category. Initially, the Jonckheere-Terpstra test supported the existence of a statistically significant (Jonckheere-Terpstra statistic = 705, one sided *p*-value of 0.0401) trend of higher median WGS-derived dLRS with higher levels of absolute HRD score difference. We note the presence of two samples (FBC050467, pointed by the purple arrow and FBC208006, pointed by the red arrow) with very high WGS-derived dLRS in the highest category of absolute HRD score difference ($10 \geq |\Delta \text{ HRD score}| \leq 32$). There was no significant trend identified in the array-derived dLRS with respect to the increasing categories of absolute HRD score difference (Jonckheere-Terpstra statistic = 686.5, one-sided *p*-value = 0.0659). **(b)** Distribution of various quality metrics for samples FBC208006 (red) and FBC050467 (purple). The following GC bias metrics were considered: AT_DROPOUT, representing how undercovered regions with GC content less than 50% are compared to the mean coverage; GC_DROPOUT, representing how undercovered regions with GC content greater than 50% are compared to the mean coverage; GC_NC_0_19, representing normalized coverage over quintile of GC content ranging from 0%–19%; GC_NC_20_39, representing normalized coverage over quintile of GC content ranging from 20%–39%; GC_NC_40_59, representing normalized coverage over quintile of GC content ranging from 40%–59%; GC_NC_60_79, representing normalized coverage over quintile of GC content ranging from 60%–79% and GC_NC_80_100, representing normalized coverage over quintile of GC content ranging from 80%–100%. We also calculated the following two WGS performance metrics: PCT_EXC_MAPQ, representing the fraction of aligned bases that were filtered out because they were in reads with low mapping quality and PCF_EXC_BASEQ, representing the fraction of aligned bases that were filtered out because they were of low base quality. We note that these two samples have a high AT_DROPOUT statistic compared to the rest of the cohort. This is accompanied by a relatively higher normalized coverage in the GC_NC_40_59, GC_NC_60_79 and the GC_NC_80_100 bins, indicative of a higher number of reads mapping in these high GC content regions. After removal of these samples from the data and rerunning the Jonckheere-Terpstra test, we now noted a lack of significant trend in the WGS-derived dLRS and absolute HRD score difference category (Jonckheere-Terpstra statistic = 591, one-sided p-value = 0.1442).

Supplementary Figure 6

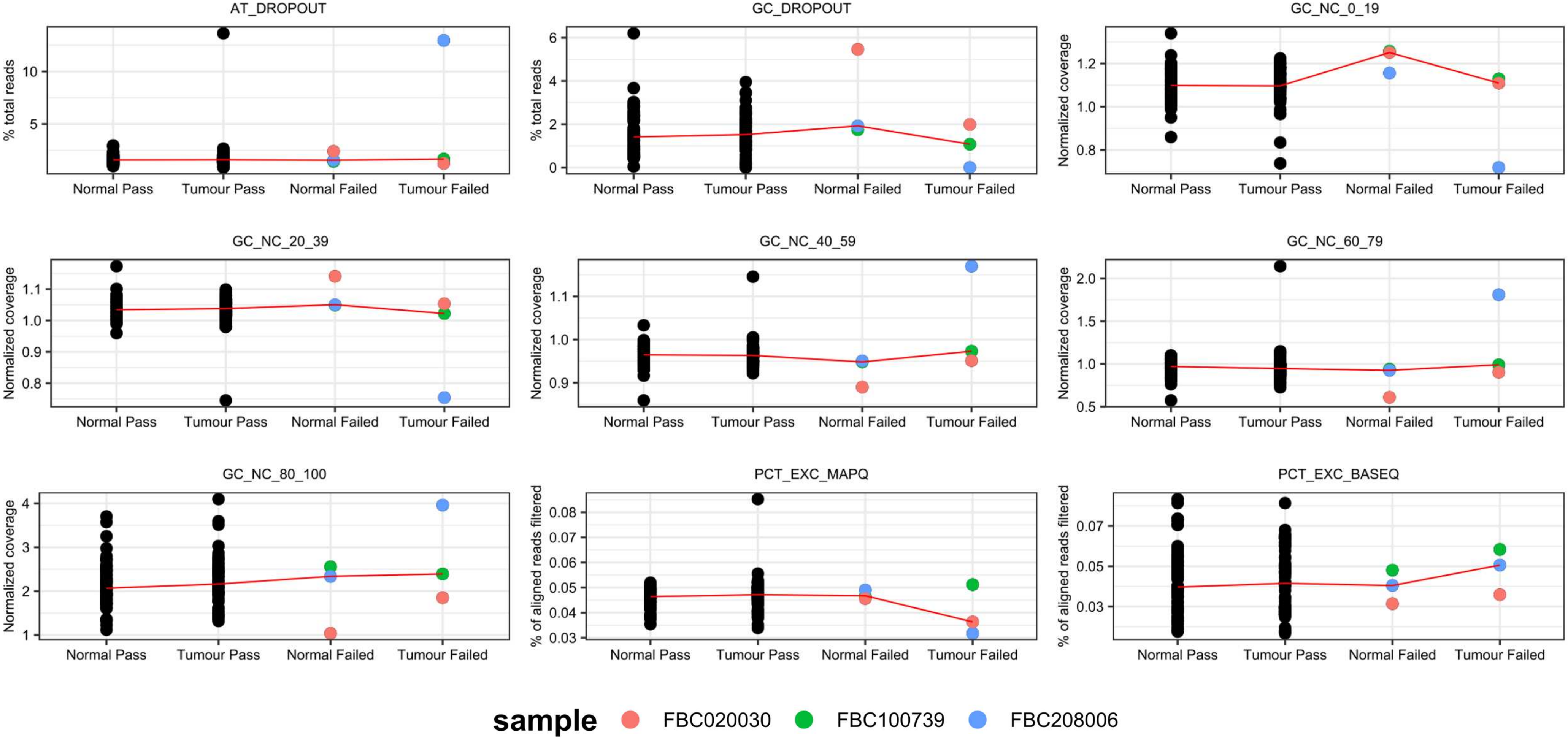

Supplementary Figure 6: WGS performance and GC bias metrics for the three samples where ascatNgs failed. We investigated various metrics representing the quality and performance of the WGS experiment to understand why ascatNgs failed to run on three downsampled WGS samples. The following GC bias metrics were considered: AT_DROPOUT, representing how undercovered regions with GC content less than 50% are compared to the mean coverage; GC_DROPOUT, representing how undercovered regions with GC content greater than 50% are compared to the mean coverage; GC_NC_0_19, representing normalized coverage over quintile of GC content ranging from 0%–19%; GC_NC_20_39, representing normalized coverage over quintile of GC content ranging from 20%–39%; GC_NC_40_59, representing normalized coverage over quintile of GC content ranging from 40%–59%; GC_NC_60_79, representing normalized coverage over quintile of GC content ranging from 60%–79% and GC_NC_80_100, representing normalized coverage over quintile of GC content ranging from 80%–100%. We also calculated the following two WGS performance metrics: PCT_EXC_MAPQ, representing the fraction of aligned bases that were filtered out because they were in reads with low mapping quality and PCF_EXC_BASEQ, representing the fraction of aligned bases that were filtered out because they were of low base quality. The red line represents trend of the median of the parameters between samples which failed to run (Failed) and those where ascatNgs successfully ran to completion (Pass). The tumour sample of FBC208006 and the normal sample of FBC020030 displayed uneven coverage, especially in regions of low (GC_NC_0_19) and high (GC_NC_60_79 and GC_NC_80_100) GC contents, which could have been aggravated with the downsampling to 15X and 10X, resulting in the failure of ascatNgs to run. Furthermore, these samples also displayed a high AT_DROPOUT (FBC208006 tumour) and GC_ DROPOUT (FBC020030 normal) metric. With respect to the WGS performance metrics, both the PCT_EXC_MAPQ and PCF_EXC_BASEQ metrics were less than 0.1% across both the samples that successfully ran or did not; this indicates that less than 0.1% of the reads were excluded due to mapping or base quality therefore quality of the mapping and base quality did not seem to be responsible in these samples failing to run on ascatNgs.

Supplementary Figure 7

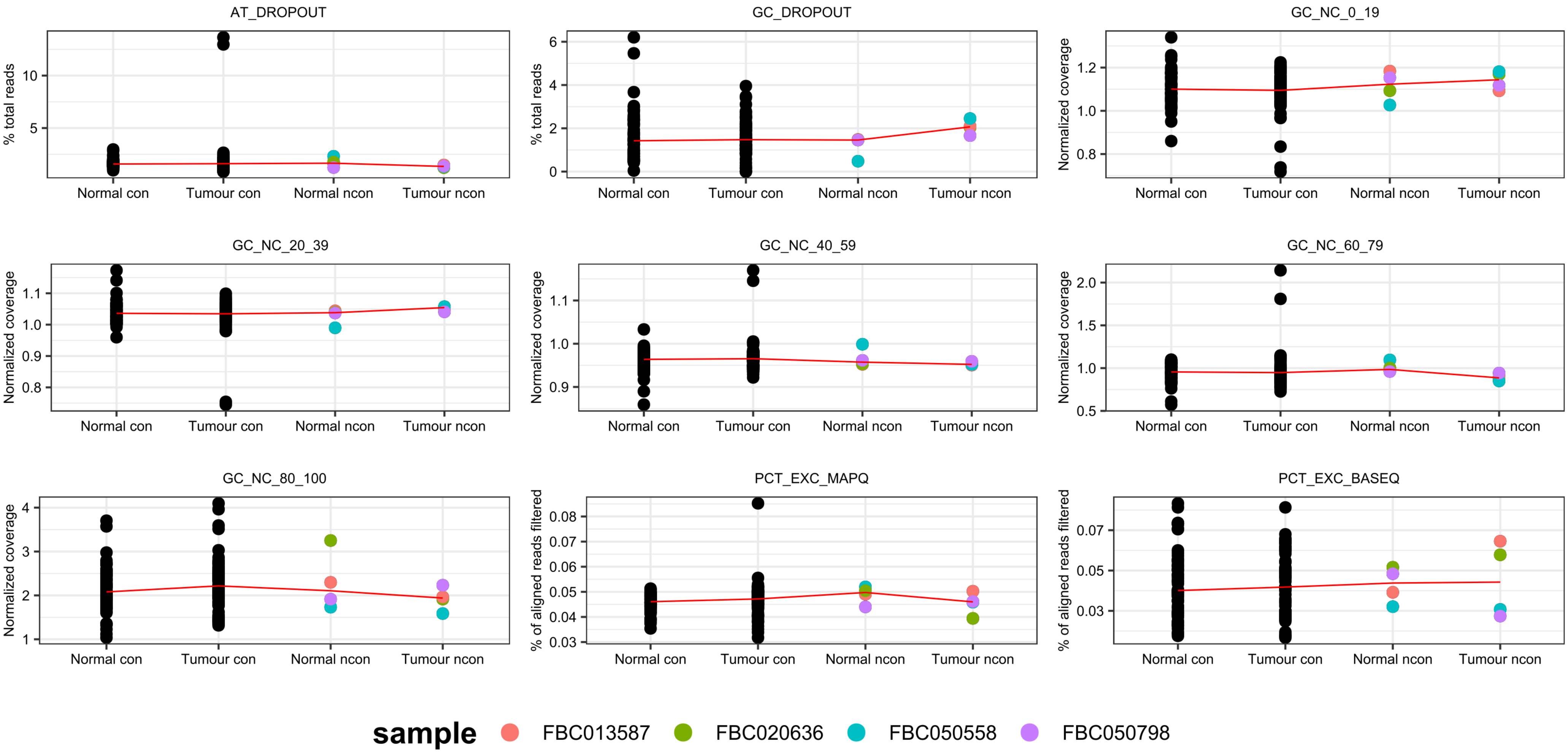

Supplementary Figure 7: WGS performance and GC bias metrics for the normal and tumour samples. To understand why four samples were non-congruently classified between the original and downsampled WGS-derived HR classification, we investigated the aforementioned GC and WGS performance metrics representing the quality and performance of the WGS experiment. The red line represents trend of the median of the parameters between samples which were congruently (con) and non-congruently (ncon) classified. Overall the majority of the GC and WGS metrics were similar between the cases that were congruently classified or not. With respect to the WGS performance metrics, both the PCT_EXC_MAPQ and PCF_EXC_BASEQ metrics were less than 0.1% across both the congruent and non-congruent samples; this indicates that less than 0.1% of the reads were excluded due to mapping or base quality therefore quality of the mapping and base quality did not seem to affect our experiment.
