# Supplementary material for: Using whole-genome sequencing data to derive the homologous recombination deficiency scores
Source: NPJ Breast Cancer. 2020 Aug 7;6:33. doi: 10.1038/s41523-020-0172-0 (PMC7414867; doi:10.1038/s41523-020-0172-0)
Supplement: Supplementary file 2 — Reporting Summary Checklist [file 41523_2020_172_MOESM2_ESM.pdf]

nature research

Corresponding author(s): Peter Simpson
Last updated by author(s): May 23, 2020

Reporting Summary

Nature Research wishes to improve the reproducibility of the work that we publish. This form provides structure for consistency and transparency in reporting. For further information on Nature Research policies, see Authors & Referees and the Editorial Policy Checklist.

Statistics

For all statistical analyses, confirm that the following items are present in the figure legend, table legend, main text, or Methods section.

n/a	Confirmed	
☐	☒	The exact sample size (*n*) for each experimental group/condition, given as a discrete number and unit of measurement
☐	☒	A statement on whether measurements were taken from distinct samples or whether the same sample was measured repeatedly
☐	☒	The statistical test(s) used AND whether they are one- or two-sided<br>*Only common tests should be described solely by name; describe more complex techniques in the Methods section.*
☐	☒	A description of all covariates tested
☒	☐	A description of any assumptions or corrections, such as tests of normality and adjustment for multiple comparisons
☐	☒	A full description of the statistical parameters including central tendency (e.g. means) or other basic estimates (e.g. regression coefficient) AND variation (e.g. standard deviation) or associated estimates of uncertainty (e.g. confidence intervals)
☐	☒	For null hypothesis testing, the test statistic (e.g. *F*, *t*, *r*) with confidence intervals, effect sizes, degrees of freedom and *P* value noted<br>*Give P values as exact values whenever suitable.*
☒	☐	For Bayesian analysis, information on the choice of priors and Markov chain Monte Carlo settings
☒	☐	For hierarchical and complex designs, identification of the appropriate level for tests and full reporting of outcomes
☐	☒	Estimates of effect sizes (e.g. Cohen's *d*, Pearson's *r*), indicating how they were calculated

Our web collection on statistics for biologists contains articles on many of the points above.

Software and code

Policy information about availability of computer code

Data collection	Fresh frozen tumours and blood from 67 familial breast cancer patients were obtained via the Kathleen Cuningham Foundation Consortium for Research into Familial Breast Cancer (kConFab), the Australian Breast Cancer Tissue Bank (ABCTB) and the Brisbane Breast Bank (BBB).
Data analysis	WGS data:<br>Sequence reads were trimmed using Cutadapt (version 1.11) and aligned to GRCh37 using BWA-MEM (version 0.7.12)<br>Duplicate alignments were marked with Picard (version 1.129, http://picard.sourceforge.net)<br>BAM files were coordinated-sorted using Samtools (version 1.3)<br>Mean coverage was determined using qCoverage (http://sourceforge.net/projects/adamajava)<br>ascatNgs (https://github.com/cancerit/ascatNgs) was used to generate allele-specific copy number profile for the WGS data using the paired tumour/normal BAM files as input<br><br>Downsampling WGS data:<br>Downsampling of the original normal and tumour BAM files was performed using the samtools (version 1.3) library function: samtools view<br><br>WGS and GC bias metrics:<br>Picard (http://broadinstitute.github.io/picard/) version 2.18.15 was used to generate WGS performance (CollectWgsMetrics) and GC bias metrics (CollectGcBiasMetrics)<br><br>Array data:<br>SNP array data was processed using the Genotyping module (v1.9.4) in GenomeStudio v2011.1 (Illumina, San Diego CA)<br>Genomestudio was used to derive B-allele frequencies (BAF) and logR ratios (LRR)<br>Copy number data was estimated using ASCAT copy number R package (Version 1.0) (https://github.com/Crick-CancerGenomics/ascat)

Measurement of agreement
R package BlandAltmanLeh (https://cran.r-project.org/web/packages/BlandAltmanLeh/index.html) version 0.3.1 was used to determine the Bland-Altman parameters
Fleiss kappa was computed using the irr package (https://cran.r-project.org/web/packages/irr/index.html) version 0.84.1
ICC3 was computed using the DescTools package (https://cran.r-project.org/web/packages/DescTools/index.html) version 0.99.28

All statistical analyses were performed in R studio version 1.1.453 using R version 3.3.3

For manuscripts utilizing custom algorithms or software that are central to the research but not yet described in published literature, software must be made available to editors/reviewers. We strongly encourage code deposition in a community repository (e.g. GitHub). See the Nature Research guidelines for submitting code & software for further information.

Data

Policy information about availability of data

All manuscripts must include a data availability statement. This statement should provide the following information, where applicable:

- Accession codes, unique identifiers, or web links for publicly available datasets
- A list of figures that have associated raw data
- A description of any restrictions on data availability

Data is available from figshare. Currently waiting for individual data DOIs to become available.

Field-specific reporting

Please select the one below that is the best fit for your research. If you are not sure, read the appropriate sections before making your selection.

☒ Life sciences ☐ Behavioural & social sciences ☐ Ecological, evolutionary & environmental sciences

For a reference copy of the document with all sections, see nature.com/documents/nr-reporting-summary-flat.pdf

Life sciences study design

All studies must disclose on these points even when the disclosure is negative.

Sample size	No sample size calculation was performed, as this study involves a retrospective analysis. 67 samples from consenting patients for which we had array and WGS data for both tumour and normal breast tissue available were selected. Given that our paper involves a direct comparison of methods, together with the results we present in the paper, our sample size was adequate to answer the questions addressed in the paper.
Data exclusions	no
Replication	Results presented here are fully reproducible when using the same softwares and data we provide.
Randomization	There was no randomization of samples/participants in experimental groups, as this was not relevant to our study.
Blinding	Investigators were not blinded to group allocation during data collection and analysis as this was not relevant for the particular type of study underlying this project.

Reporting for specific materials, systems and methods

We require information from authors about some types of materials, experimental systems and methods used in many studies. Here, indicate whether each material, system or method listed is relevant to your study. If you are not sure if a list item applies to your research, read the appropriate section before selecting a response.

Materials & experimental systems

n/a	Involved in the study	
☒	☐	Antibodies
☒	☐	Eukaryotic cell lines
☒	☐	Palaeontology
☒	☐	Animals and other organisms
☐	☒	Human research participants
☒	☐	Clinical data

Methods

n/a	Involved in the study	
☒	☐	ChIP-seq
☒	☐	Flow cytometry
☒	☐	MRI-based neuroimaging

Human research participants

Policy information about studies involving human research participants

Population characteristics	The 67 patients included carriers of pathogenic germline mutations in BRCA1 (n = 18) or BRCA2 (n = 21) and 28 tumours from high risk individuals from breast cancer families not attributed to BRCA1 or BRCA2 germline mutations (non-BRCA1/2)
Recruitment	High-risk familial breast cancer patients, where both tumour and normal DNA was available, and who consented to participating in the research were recruited for this study. The patients were recruited by three tissue banks (below) and consented to the use of their biospecimens for research purposes. Tissue banks: Kathleen Cuningham Foundation Consortium for Research into Familial Breast Cancer (kConFab), the Australian Breast Cancer Tissue Bank (ABCTB) and the Brisbane Breast Bank (BBB).
Ethics oversight	Work performed was covered by Human Research Ethics Committee approval from the University of Queensland (2005000785) and QIMR Berghofer Human Research Ethics Committee (P3527)

Note that full information on the approval of the study protocol must also be provided in the manuscript.
